# Malignant chondroid syringoma: A systematic review

**DOI:** 10.1002/ski2.144

**Published:** 2022-07-10

**Authors:** Alina G. Zufall, Erica J. Mark, Alejandro A. Gru

**Affiliations:** ^1^ Department of Pathology University of Virginia Charlottesville Virginia USA

## Abstract

Malignant Chondroid Syringomas (MCS) are very rare malignant tumours arising from cutaneous sweat glands, with only 51 reported cases in the literature. These tumours can metastasize and cause death if not treated adequately. While there are histological criteria to diagnose MCS tumours, there are no established criterion to determine which tumours are more or less likely to metastasize. A systematic review was performed to establish if any features of the primary MCS tumour are associated with risk of metastasis or patient mortality, as well as the efficacy of common treatment options. The literature search was performed using the Ovid Medline and Web of Science databases from inception through March 2020. This yielded 47 case reports corresponding to 51 unique patients. Statistical analysis of the collected data revealed none of the commonly accepted malignant histopathologic findings (including nuclear atypia and/or pleomorphism, mitotic figures, an infiltrative growth pattern, presence of satellite nodules, necrosis, and vascular and/or perineural invasion) of the primary tumour to be significantly more associated with metastatic risk or death. However, gross characteristics of the tumour, including size (greater than 5 cm) and truncal location of the primary lesion, were found to be associated with a higher risk of metastasis. The most effective treatment modality was wide local excision. Overall, primary MCS tumours, especially those greater than 5 cm or located on the trunk, should be treated with a wide local excision and followed closely to confirm no lesion recurrence or distant metastasis.

## Graphical abstract

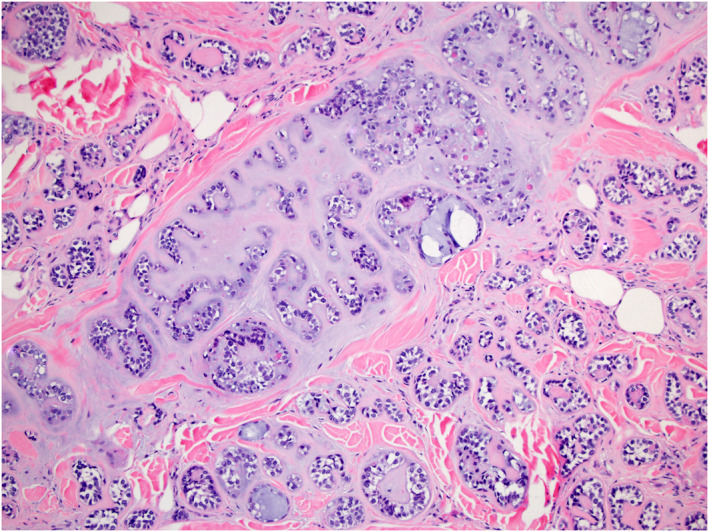

1

**What's already known about this topic?**

- Malignant chondroid syringoma (MCS) is an incredibly rare condition with 51 reported cases in the literature
- No established criterion exist to determine which MCS tumours are more or less likely to metastasize

**What does this study add?**

- None of the commonly accepted malignant histopathologic findings (including nuclear atypia and/or pleomorphism, mitotic figures, an infiltrative growth pattern, presence of satellite nodules, necrosis, and vascular and/or perineural invasion) of the primary tumour are significantly more associated with metastatic risk or death
- Gross characteristics of the tumour, including size (greater than 5 cm) and truncal location of the primary lesion, were found to be associated with a higher risk of metastasis
- The most effective treatment for preventing metastasis and recurrence was wide local excision

## 2 INTRODUCTION

Chondroid Syringomas, or cutaneous mixed tumours, are benign tumours that arise from eccrine and/or apocrine glands. The term “Chondroid Syringoma” was first used by Hisch and Helwig in 1961 to describe these relatively rare tumours.^1^ They often arise as asymptomatic, firm subcutaneous nodules and are usually present for several years with little to no change in size. Histopathologic criteria include: (1) nests of cuboidal or polygonal cells, (2) tubuloalveolar structures, (3) ductal structures, and (4) a matrix of varying appearances (chondroid or hyaline material). There can be keratinous cysts lined with squamoid cells present.^1^ Immunohistochemistry can be helpful, usually showing expression of a combination of different epithelial and mesenchymal markers: cytokeratins, epithelial membrane antigen (EMA), S‐100, vimentin, carcinoembryonic antigen (CEA), and blood group antigen H.^2^

Malignant chondroid syringomas (MCS) are much rarer than their benign counterpart, with only 51 reported cases in the literature. The histologic criteria for malignancy have been somewhat arbitrary, as histologically benign appearing tumours have recurred or metastasized. Histopathologic criteria for malignancy incudes: nuclear atypia and/or cellular pleomorphism, presence of frequent mitotic figures, an infiltrative growth pattern, presence of satellite nodules, necrosis, and vascular and/or perineural invasion.^3^

The purpose of this systematic review was to determine if any features of the primary MCS (including location, size, and histological features) were associated with increased risk of metastasis or patient mortality secondary to their disease. Secondarily, we reviewed common treatments of MCS tumours to determine which were the most effective at preventing recurrence or metastasis. While many MCS case reports in the literature include a review of several MCS cases, this is the largest review to date, offering valuable insight to the common characteristics and behaviour of these rare tumours.

## 3 MATERIALS AND METHODS

**FIGURE 1:**
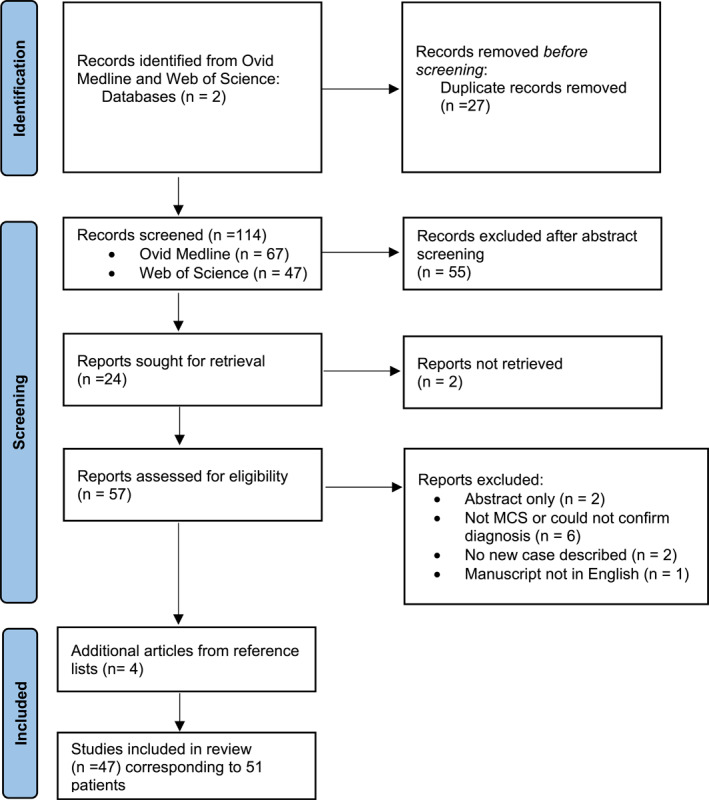
Systematic review overview flow diagram

The data for this review was extracted and assessed by the reviewers using the PRISMA guidelines (Figure 1).^4^ No methods were used to assess the risk of bias in study design or reporting, as this review consisted only of case reports and small case series. This systematic review was conducted using the Ovid Medline and Web of Science databases from inception through March 2021. The search terms “malignant chondroid syringoma” and “malignant mixed tumour AND cutaneous” were used in both databases. “Malignant cutaneous mixed tumour” was also used in Ovid Medline. Web of Science searches were limited to English language only. Review papers were used to find any additional case reports missed in the initial literature search.

Reports were included in this review if there was a new patient case and clinical and histological evidence of MCS. The diagnosis of MCS was confirmed by a dermatopathologist based on review of each case report, which included the lesion's clinical history, histological description, and immunohistochemistry results (if available) provided in each report. Retrieval of clinical information obtained in the case reports and small series included: sex, age, ethnicity, past medical history, description of the clinical lesions, imaging, histopathology, treatment, and disease outcome. Malignant histopathologic findings, in accordance with the MCS review by Bates and Baithun's in 1998, were tabulated for each case.^3^ These included: nuclear atypia and/or pleomorphism, mitotic figures, infiltrative growth pattern, presence of satellite nodules, necrosis, and vascular and/or perineural invasion. Mitotic figures were described as either rare or frequent. Sub‐categories were limited by the availability of histopathologic description written in each manuscript. Histological images were not used to add or subtract additional information, as the quality was often poor or there were only one or two frames shown.

All statistical analyses were performed using R (ver. 4.0.2) and RStudio (ver. 1.2.5033).^5^ Descriptive statistics, including frequencies and percentages, were calculated for all variables except age, months until primary lesion was treated, lesion size, and number of years until metastasis. For these characteristics, median and interquartile range (IQR) were reported due to the non‐parametric distribution. Chi‐square tests were used to compare primary tumour features and their association with local recurrence and/or metastasis. This included comparing across different malignant histological features, location, and treatments of the primary tumour. The Wilcoxon Rank Sum Test was used to compare the average size of lesions that recurred and/or metastasized to those that did not.

## 4 RESULTS

In this review, 114 related articles were identified using the search criteria discussed above (Figure 1). Twenty‐seven duplicate records were then removed. After the initial abstract screening, 57 articles remained, 11 of which were then removed because they did not meet inclusion criteria. Using reference lists of MCS review publications, four additional articles were discovered and included in this review. In total, 47 reports were used, which corresponded to 51 unique patients. All manuscripts included were single case reports or small case series; there were no cohort studies or randomized‐controlled trials found. There was a duplicate patient across two separate case reports.^6^, ^7^

A summary of data from reported cases of MCS's in the literature is detailed in Table 1. The median age of MCS diagnosis was 53 (IQR = 33) with a range (R) of ages 9–92 (Table 2). Many of these lesions were not treated for months following initial diagnosis (median = 18, IQR = 28.5, *R* = 3–240). Common patient concerns prior to initial medical evaluation included growth of the lesion (25/51, 49%) and/or antecedent trauma at the site (6/51, 12%). All lesions were determined to be malignant either by their malignant histopathologic features or the presence of metastatic disease, even if the primary lesion was considered benign at initial diagnosis. Most MCS tumours were asymptomatic (32/51, 63%), nodular (39/51, 76%) with no overlying skin changes (28/51, 55%), and located on the extremities (26/51, 51%). The median size of MCS's was 4 cm (IQR = 3.5, *R* = 1–10). The most common malignant histological feature was nuclear atypia and/or pleomorphism (41/51, 80%) followed by the presence of mitotic figures (35/51, 69%). Rare mitoses (19/35, 54%) were more commonly noted than frequent ones (15/35, 43%). Infiltration of the stroma or satellite nodules (14/51, 27%), necrosis (11/51, 22%), and vascular and/or perineural invasion (7/51, 14%) were less common malignant histopathologic features.

**TABLE 1:** Summary of data from reported cases

Publication (Author, year)	Gender	Primary lesion		Malignant pathological findings						Number of local recurrences (over years form primary lesion)	Metastasis, years after primary lesion first treated (location)	Treatment			Outcome
		Location	Size (longest length in cm)	Nuclear atypia/pleomorphism	Vascular and/or perineural invasion	Infiltration through the fibrous mantle	Mitotic Figures (none, rare, frequent)	Necrosis	Satellite nodules			Primary lesion	Local recurrence	Metastasis	
1) Agrawal et al., 1998^6^	Female	Scalp	6	Yes			Rare			2 (1)	Node, 1 (occipital)	S (SE)	S (WLE)	RT	Clear
2) Mishra and Agarwal, 1998^7^															
Araujo et al, 2012^8^	Female	Scalp		Yes	Yes	Yes		Yes		0	No	S (WLE), complement therapy unspecified			Clear
Barnett et al, 2000^9^	Male	Foot	1	Yes		No	Frequent	Yes		1 (4)	No	S (SE)	S (WLE), RT		Clear
Bates and Baithun, 1998^3^	Male	Toe	3	Yes		Yes	Rare			0	No	S (AP)			Clear
Bates and Baithun, 1998	Male	Foot	2	Yes		Yes	Rare			0	No	S			Clear
Bates and Baithun, 1998	Female	Finger	2.2	Yes		No	Rare	Yes	Yes	0	No	S			Clear
Chauvel‐Picard et al, 2018^10^	Female	face	10	Yes		Yes	Rare			0	No	S (WLE)			Clear
Clark, 1987^11^	Female	Thigh	8	Yes		Yes	Rare			0	No	S (WLE)			
Devine et al, 1981^12^	Male	Foot	8.5	Yes			Rare			0	Organ, 1.5 (bone, muscle, soft tissue, lung, skin)	S		S (AP), RT	Death
Devine et al, 1981	Male	Thigh	3.5	Yes	Yes	Yes	Rare		Yes	0	No	S (WLE)			Clear
Dissanayake and Salm, 1980	Female	Sacram	8	Yes			Frequent	Yes		0	Organ, N/A (lung)	S			Death
Dissanayake and Salm, 1980^13^	Male	Foot		Yes			Rare				Organ, 0 (bone and lung)	S			Lost to follow up
Favareto et al, 2020^14^	Female	Knee	2							0	Node, 4 (inguinal and iliac)	S (SE)		S, RT	Clear
Fernandez‐Flores and Cassarino, 2017^15^	Male	Scalp	1.2	Yes		Yes		Yes			No	S (WLE)			
Gupta et al, 1982^16^	Male	Leg									Organ, 1 (skin)	S		S (WLE), CT, RT, S(AP)	Clear
Henriquez et al, 2000^17^	Male	Finger	2	Yes			Frequent	Yes		0	Node, 0 (epitrochlear)	S (AP)		CT	Clear
Hilton and Blackwell, 1973^18^	Female	arm	1.3	Yes		No	Frequent			1 (16)	Node, 11 (axillary)	S (WLE)		S	Clear
Hong et al, 1995^19^	Female	Suprapubic area	6	Yes			Frequent			1 (1)	No	S (SE)	S (SE), RT		Clear
Ishmura et al, 1983^20^	Male	Back	7	Yes			Rare			0	Organ, 7 (spine, bone, liver, lung)	S		S	Death
Ka et al, 2016^21^	Male	Shoulder	4.5	Yes	Yes	Yes		Yes		0	Node, 0 (axillary)	CT			Death
Kiely et al, 1997^22^	Female	Hand									Organ, 17 (lung)	S		None	Stable disease
Kothiya et al, 2017^23^	Female	Thigh		Yes			Frequent			0	Organ, 3 (lung)	S		None	Palliative care
Krishnamurthy et al, 2015^24^	Male	Ear	3	Yes			Rare	Yes		0	Node, 0 (cervical)	S (WLE), RT			
Lal et al, 2018^25^	Male	Back	3		Yes						No	S (WLE)			
Malik et al, 2013^26^	Female	Scalp		Yes			Rare			0	No	S (WLE)			Death
Mathiasen et al, 2005^27^	Female	face	10	Yes	Yes	Yes				1 (2)	No	S (WLE), RT	S (WLE)		Clear
Matz et al, 1969^28^	Female	Scalp	5	Yes			Rare			2 (1.5)	Organ, 1.5 (posterior cervical, skin, lungs)	S (WLE)	S (WLE), local infusion with MTX, RT	RT	Death
Menendez et al, 2015^29^	Female	Abdomen								2 (15)	Organ, N/A (liver, kidney, spine)	S (SE)	S (SE)	S, RT	Stable disease
Metzler et al, 1996^30^	Male	Hip	4	Yes			Variable	No		1 (0.16)	Node, 0.5 (hip)	S (SE)	S (WLE)		
Nakanishi, 2018^31^	Male	Finger	1	Yes						0	No	S (WLE)			Clear
Nel et al, 2019^32^	Male	Scalp	4	Yes	No		Frequent			0	No	S (WLE)			Clear
Nel et al, 2019	Female	Thigh	8	Yes			Frequent	Yes		0		S (WLE)			Lost to follow up
Nguyen and Cassarino, 2017^33^	Female	Fingre	1.5							1 (20)	No	S	S (WLE)		Clear
Nicolaou et al, 2001^34^	Male	Hand	4.5	Yes			Frequent			0		S (WLE)			
Panagopoulos et al, 2019^35^	Male	Chest						Yes		0		S			
Redono et al, 1982^36^	Female	Foot								5 (N/A)	Node, N/A (inguinal)	S		CT	Condition worsened
Requena, 2013^37^	Male	face		Yes					Yes		No	S (WLE), RT			
Rosborough 1963^38^	Female	arm	3	Yes			Frequent			1 (1)	Node, 1 (axillary)	S (SE)	S (WLE)	S	Clear
Sanchez Herreros et al, 2011^39^	Female	face	2.5	Yes			Rare			0	No	S (MMS)			Clear
Sanchez Yus et al, 1988^40^	Female	face	3	Yes			Frequent			0	No	S			Clear
Scott and Metcalf, 1988^41^	Male	Neck	5	Yes			Frequent			0	Node, N/A (cervical)	S (WLE)		RT	Lost to follow up
Shashikala et al, 2004^42^	Female	Scalp	5	Yes	Yes		Rare				No	S			
Shobhanaa et al, 2016^43^	Female	Scalp, neck	Primary lesion not described							1 (N/A)					
Shvili and Rothem, 1986^44^	Female	Glutaeus	5	Yes			Frequent			0	Organ, 0 (lung, liver, kidney, thyroid, brain, vertebrae, bone)	S (WLE)		CT, RT	Death
Steinmetz et al, 1990^45^	Male	Back	4	Yes			Frequent			0	Organ, 0 (liver, lungs brain)	S (WLE)		None	Death
Stromberg, 1991^46^	Male	face	3	Yes			Rare	Yes		0	No	S (WLE)			
Sun et al, 1996^47^	Male	Foot				Yes				1 (6)	Organ, 7 (bone, brain)	S (WLE)	S (WLE)	S (AP), RT (palliative)	Death
Takakashi et al, 2004^48^	Female	Toe	2.5	Yes			Rare			2 (1.5)	No	S (SE)	S (AP)		Clear
Tural et al, 2013^49^	Female	face		Yes	Yes		Rare			1 (1)	No	S (WLE)			Clear
Watson et al, 1991^50^	Female	Foot		Yes		Yes	Frequent			0	No	S (AP)			
Webb and Scott, 1975^51^	Female	Thigh	8	Yes			Rare			1 (1)	Node, 1 (inguinal)	S (SE)	S (WE)	S	Clear

Abbreviations: AP, amputation; CT, chemotherapy; MMS, Mohs Micrographic Surgery; MTX, methotrexate; RT, radiation therapy; S, surgery; SE, simple excision; WLE, wide local excision.

**TABLE 2:** Summary of malignant chondroid characteristics in the literature

MCS characteristics	Median (IQR), range
Age of patient when primary lesion arose	53 (33), 9–92
Months until primary lesion treated	18 (28.5), 3–240
Lesion size (longest length in cm)	4 (3.5), 1–10
Number of years until metastasis	1 (3.25), 0–17

	Number of cases (%)
History	
Increasing in size	25 (49)
Trauma at site	6 (12)
Associated symptoms	
Asymptomatic	32 (63)
Painful	14 (27)
Location	
Extremities	26 (51)
Head/neck	17 (33)
Trunk	8 (16)
Primary morphology	
Nodule	39 (76)
Papule	2 (4)
Cyst	2 (4)
Plaque	1 (2)
Secondary morphology	
No overlying skin changes	28 (55)
Ulceration	7 (14)
Malignant histological features	
Nuclear atypia and/or pleomorphism	41 (80)
Mitotic Figures	35 (69)
Rare	19 (37)
Frequent	15 (29)
Variable	1 (2)
Infiltration or satellite nodules	14 (27)
Necrosis present	11 (22)
Vascular and/or perineural invasion	7 (14)
Number of local recurrences	
0	28 (55)
1	12 (24)
>1	3 (6)
Metastasis	
None	24 (47)
Local lymph node (s)	11 (22)
Organ (s)	12 (24)
Lung	8 (67)
Bone	6 (50)
Liver	4 (33)
Brain	3 (25)
Skin	2 (17)
Spine	2 (17)
Kidney	2 (17)
Thyroid	1 (8)
Treatment	
Primary tumour	50 (98)
WLE	19 (38)
S (unspecified)	14 (28)
SE	9 (18)
AP	3 (6)
S + RT	3 (6)
MMS	1 (2)
Local recurrence	12 (24)
WLE	7 (58)
S + RT	3 (25)
SE	1 (8)
AP	1 (8)
Metastasis	18 (35)
S (mets)	4 (22)
S (mets) + RT	4 (22)
RT	3 (17)
CT	2 (11)
CT + RT	1 (6)
S(mets) + CT + RT	1 (6)
No treatment	3 (17)
Outcome	
Clear	23 (45)
Death	9 (18)
Condition stable	2 (4)
Condition worsened (including palliative care)	2 (4)
Lost to follow up	3 (6)

Abbreviations: AP, amputation; CT, chemotherapy; mets, metastasis; MMS, Mohs Micrographic Surgery; MTX, methotrexate; RT, radiation therapy; S, surgery; SE, simple excision; WLE, wide local excision.

MCS tumours can be aggressive, with 45% (23/51) of reported MCS tumours locally recurring and 52% (27/51) metastasizing. Local recurrence did not predict future metastasis, with 59% (10/17) of tumours metastasizing after local recurrence and 44% (11/25) metastasizing without any evidence of local recurrence. Twelve lesions locally recurred once and only three lesions locally recurred more than once. Twenty‐three primary MCS tumours metastasized, with 11 metastasizing to local lymph node(s) and 12 metastasizing to distant organ(s). The most common site of organ metastasis was the lungs (8/12, 67%). Other locations included bone (6/12, 50%), liver (4/12, 33%), brain (3/12, 25%), skin (2/12, 17%), spine (2/12, 17%), kidney (2/12, 17%), and thyroid (1/12, 8%). The most common treatment for the primary tumour was wide local excision (WLE) (19/50, 38%) followed by a standard excision (SE) (9/50, 18%). In 14 cases, the type of surgery was unspecified. Radiation therapy (RT) was rarely added to the treatment of primary lesions (3/50, 6%). A full amputation of the affected limb was performed in three cases (6%). Mohs Micrographic Surgery (MMS) was only performed in one case (2%). WLE was also the most common treatment for local recurrences (7/12, 58%), with adjunct local RT added to surgery in three cases (25%). For metastatic disease, surgery, RT, and chemotherapy (CT) were all utilized. Surgery and surgery plus RT were the most commonly used treatment for metastases (8/18, 44%).

**FIGURE 2:**
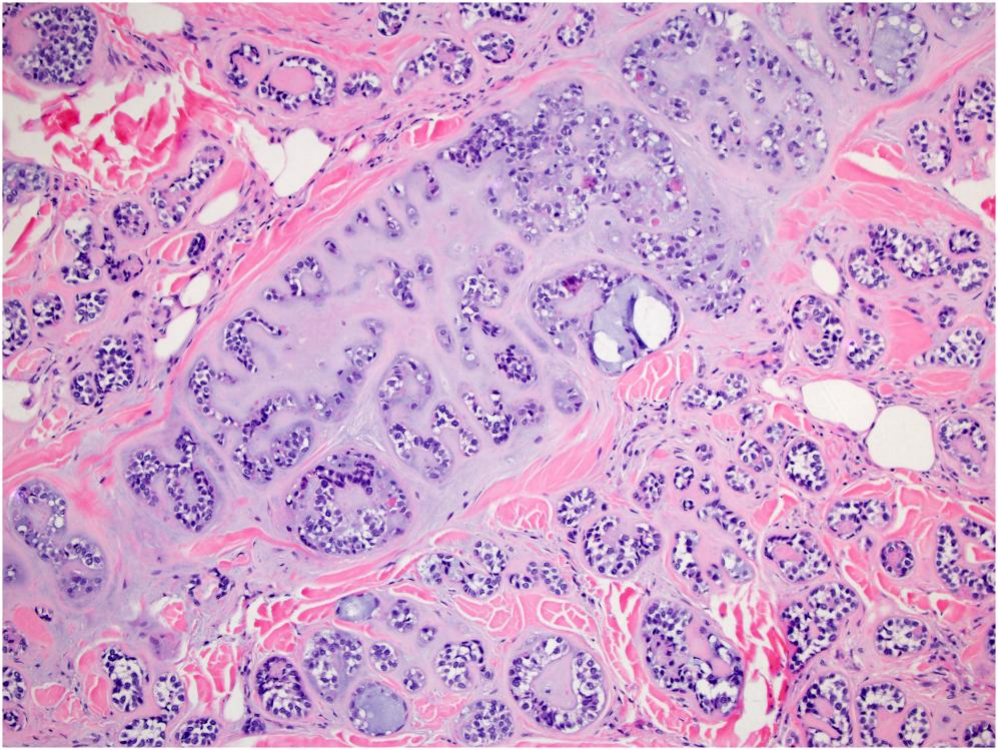
Histopathologic changes show a malignant adnexal tumour with a rich chondroid matrix (H&E 100x)

**FIGURE 3:**
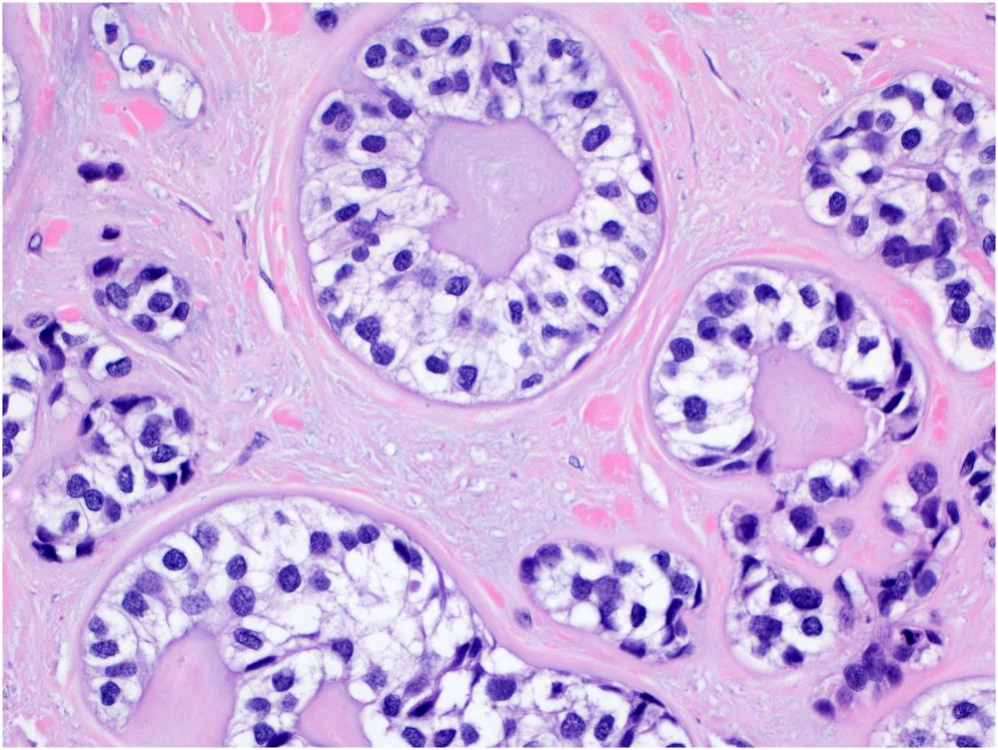
The malignant glandular elements show ductular structures with abundant clear cytoplasm and hyperchromatic nuclei (H&E 400x)

**FIGURE 4:**
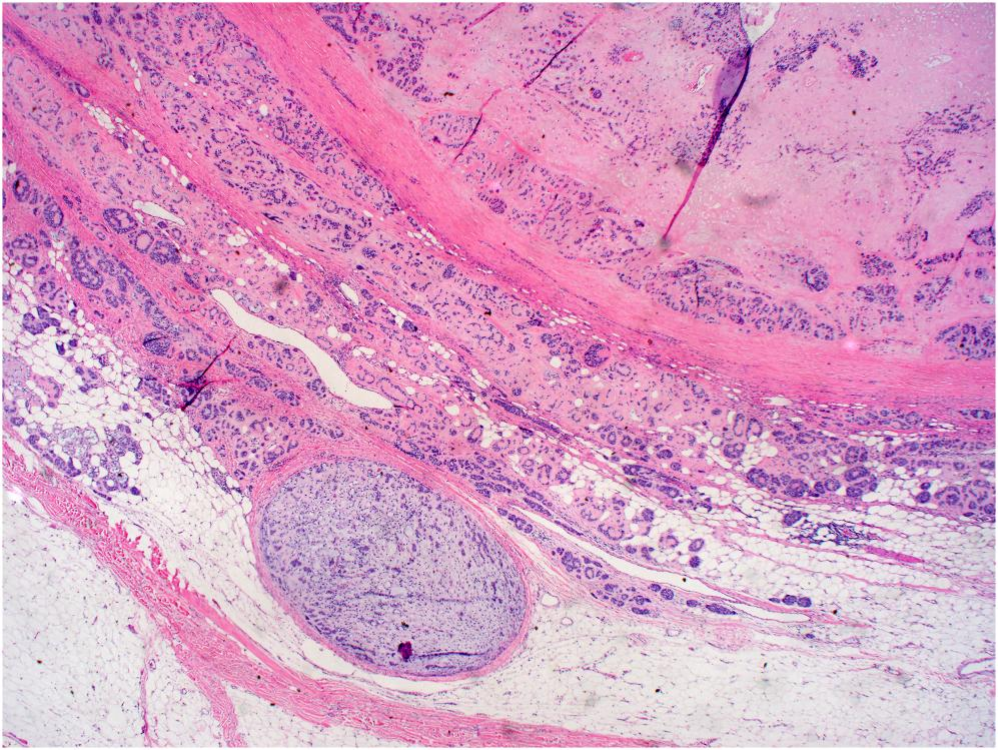
This malignant mixed tumour shows invasion and infiltration into the subcutaneous tissue (H&E 40x)

**FIGURE 5:**
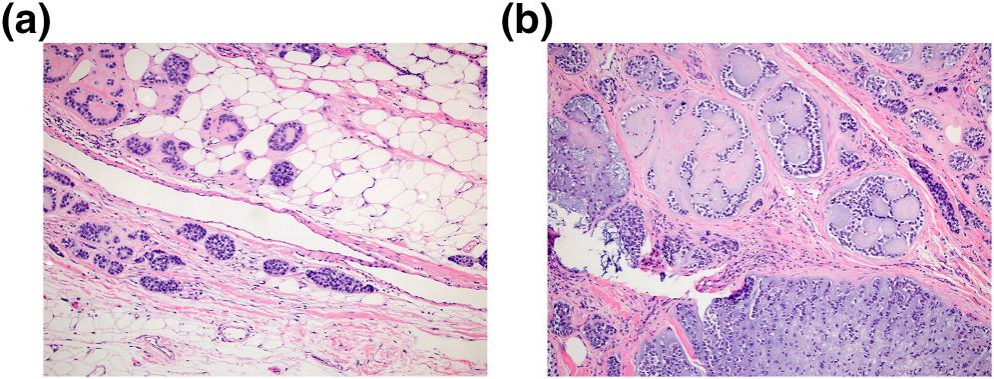
(a and b): Malignant mixed tumour shows invasion and infiltration of malignant glandular structures into the subcutaneous deep tissue. Superficial necrosis is present. (H&E 100x (a) and 200x (b))

The key histopathologic features of MCS and their association with metastatic risk and death are summarized in Table 3 and Figures 2, 3, 4, 5. The median number of years from the treatment of the first lesion to death was 3.83 (IQR = 7.46) with a range of 0–12 years. No specific histological features of the primary MCS tumour were found to be significantly associated with later development of metastasis or death from the patient's disease, though the highest percentage (48.5%) of primary tumours with mitotic figures present on histology eventually went on to metastasize. This was specifically true for lesions with frequent mitotic figures, with 61.5% metastasizing compared to only 36.8% with rare mitotic figures. While MCS tumours are commonly located on the extremities, truncal lesions had a higher metastatic risk (6/8, 75%) and were more frequently associated with disease‐specific mortality (4/5, 80%, *p* = 0.02). Tumours on the head and neck were least commonly associated with metastasis (6/17, 35.3%). The median size of lesions that metastasized was only slightly larger (5 cm) than those that did not (3 cm) and was not significant in determining metastatic risk. However, there was a significant difference in the median size of lesions in patients with fatal disease (7.0 cm) compared to those with non‐fatal disease (2.8 cm) (*p* = 0.01).

**TABLE 3:** Features of primary tumour associated with metastasis and death

Features of primary tumour	Number of cases with metastasis/Total in that category (%)	*p* value^a^	Number of deaths/Total in that category (%)	*p* value^b^
Malignant histological features		0.17		1
Mitotic Figures Present	16/33(48.5)	0.29	7/24 (29.2)	1
Frequent	8/13 (61.5)		3/10 (30.0)	0.02*
Rare	7/19 (36.8)		4/14 (28.6)	
Necrosis present	4/9 (44.4)		2/6 (33.3)	
Nuclear atypia and/or pleomorphism	17/39 (43.6)		8/28 (28.6)	
Infiltration or satellite nodules	2/13 (15.4)		2/9 (22.2)	
Vascular and/or perineural invasion	1//7 (14.3)		1/5 (20.0)	
Location of primary lesion		0.07		
Trunk	6/8 (75.0)		4/5 (80.0)	
Extremity	17/26 (65.4)		3/17 (17.6)	
Head/neck	6/17 (35.3)		2/10 (20.0)	

	Median size for cases without, with metastasis (cm)	*p*‐value^a^	Median size for cases resulting in clearance versus death	*p*‐value^b^
Size of lesion	3, 5	0.10	2.8, 7.0	0.01*

^a^ *p*‐value determined by the chi‐square test.

^b^ *p*‐value determined by the Wilcoxon signed‐rank test.

*p‐value significant, <0.05.

The efficacy of different treatment options for the primary tumour is summarized in Table 4. SE's were significantly less effective than WLE's (including amputations) and WLE's plus radiation therapy; 89% (8/9) of primary tumours treated with SE locally recurred, while only 20% (3/15) of cases of treated with a WLE locally recurred (*p* = 0.002). SE were also less effective at preventing metastasis, with 63% (5/8) of cases metastasizing after a SE versus 36% (7/19) after a WLE; however, this difference did not reach significance. Radiation therapy added to the WLE did not seem to significantly reduce the chance of local recurrence or metastasis, though only three primary tumours were treated with additional radiation therapy, with one of these cases not reporting on local recurrence.

**TABLE 4:** Treatment of the primary lesion and chance of recurrence or metastasis

Treatment of primary lesion	Local recurrence (%)	*p*‐value	Metastasis (%)	*p*‐value
Standard excision	8/9 (89)	0.002*	5/8 (63)	0.48
Wide‐local excision (including amputation)	3/15 (20)		7/19 (36)	
Wide‐local excision + radiation therapy	1/2 (50)		1/3 (33)	

*Note*: *p*‐values determined with a 3 × 2 chi‐square test.

*p‐value significant, <0.05.

## 5 DISCUSSION

Malignant chondroid syringomas are rare adnexal tumours with very few reported cases in the literature. The histopathologic criteria currently used to define malignancy are arbitrary, vague, and poorly reproducible, given the uncommon nature of this diagnosis. Because these tumours can metastasize, even when the primary tumour appears histologically benign, it is important to determine if any features of the primary tumour are associated with metastasis of these lesions and/or disease‐specific mortality. The efficacy of different treatments of the MCS tumours was also analyzed based on the outcomes of reported cases.

MCS tumours often present as a non‐specific painless subcutaneous nodule, and therefore are usually of little concern to the patient and go untreated for months to years. Lesion growth or pain are typically the presenting symptoms that prompt patients to seek evaluation. Work‐up of these tumours always involves a tissue biopsy (often with immunohistochemical analysis) but may also involve fine needle aspiration or imaging. According to Shobhanaa et al., fine‐needle aspiration cytology (FNAC) may be helpful in confirming a diagnosis of MCS when it is unclear.^8^ Features suggesting malignancy on FNAC include: increased cellularity, a haemorrhagic background, and discohesive pleomorphic epithelial cells in ill‐forming cords.^7^ Imaging (including ultrasound, x‐ray, and computed tomography) was not often used to work‐up the primary tumour itself, but rather to evaluate for lymphadenopathy and/or metastasis if malignancy was suspected. Nicolaou et al. suggested that while Magnetic Resonance Imaging (MRI) may yield non‐specific findings, it can help to show the anatomical extent of the lesion, including location, tissue of origin, and depth of invasion.^9^

Regarding prognosis, 18% (9/51) of patients reportedly died from their disease. This is lower than the mortality rate found by Mathiasen et al. in 2005, who reported that 27% (8/30) of patients died from their MCS tumour.^10^ While only 35% of MCS tumours metastasized, this risk is high enough to necessitate frequent monitoring of these patients for local recurrence, lymphadenopathy, or distant metastasis. No malignant histological findings in the primary tumour were found to be significantly more associated with lesion metastasis or patient death, though a high proportion of primary tumours with frequent mitotic figures on histology eventually went on to metastasize. Location of the primary lesion was found to impact mortality. Significantly more patients died from their disease when the primary lesion was on the trunk than any other location. Truncal lesions also had the highest rate of metastasis (75%), though location of lesion and metastatic risk bordered significance. While MCS tumours were most commonly located on the extremities, these were least likely to result in patient death even though the metastasis rate was high (65.4%). Large lesion size was also significantly associated with patient mortality, with the median size of a primary lesion resulting in death being 7 cm compared to 2.8 cm for non‐fatal lesions. Larger lesions were also more likely to metastasize, though this was not significant. Taken together, providers may want to offer closer follow‐up to patients found to have MCS tumours that are large (greater than 5 cm), located on the trunk, and/or have frequent mitotic figures present on histological examination.

According to our review, and in agreement with previous published reviews, wide local excision is the most effective initial treatment for MCS tumours, likely due to the presence of microscopic satellite nodules.^11^ Standard excisions without wide margins were much more likely to lead to local tumour recurrence and metastasis than wide‐local excisions. One primary tumour was treated with MMS and there was no local recurrence or metastasis of this lesion. Given the infiltrative nature of the tumour and presence of satellite nodules, MMS is likely a promising treatment option and should be considered.^12^ Our results did not demonstrate a significant positive impact on concomitant RT for the primary tumour, though only three cases used this treatment. RT was more commonly used after metastasis had occurred. Efficacy of the various treatments of metastases was not assessed.

This review has some limitations. First, there are no prospective studies analyzing MCS tumours due to the rarity of the disease, so this analysis is limited to case reports and case series. The authors were also limited by the information documented by the authors of each manuscript. Information was especially limited in the histological descriptions provided. The absence of certain characteristics could rarely be confirmed, as the omission of that information in the description was not enough to confirm its absence. This limited analysis to only the presence (not absence) of certain histological characteristics. Tumour outcomes (including recurrence and metastasis), treatment, and patient outcomes (including death or clearance) was sometimes not mentioned in the manuscript, which limited analysis in these cases. Three patients were lost to follow up.

## 6 CONCLUSION

MCS tumours are rare lesions that have the potential to metastasize and lead to patient death. Careful monitoring of patients diagnosed with these tumours is paramount, especially if the initial lesion is large and located on the trunk. No histological features were more predictive of metastasis or death, however frequent mitotic figures were more commonly associated metastasis than any other feature. WLE is the most effective treatment for primary MCS tumours, however MMS can be considered given its theoretical potential benefit.

## AUTHOR CONTRIBUTION

**Alina G. Zufall**: Writing – review & editing; Writing – original draft. **Erica J. Mark**: Writing – review & editing. **Alejandro A. Gru**: Writing – review & editing.

## CONFLICTS OF INTEREST

There are no conflicts of interest to disclose.

## ETHICS STATEMENT

Not applicable.

## Data Availability

The data that supports the findings of this study are available in Table 1 of this article.
